# Help-seeking behavior, treatment barriers and facilitators, attitudes and access to first-line treatment in German adults with obsessive-compulsive disorder

**DOI:** 10.1186/s12888-025-06655-0

**Published:** 2025-03-11

**Authors:** Katharina Bey, Severin Willems, Anna Lena Dueren, Alexandra Philipsen, Michael Wagner

**Affiliations:** 1 Department of Psychiatry and Psychotherapy, University Hospital Bonn, Bonn, Germany; 2 Department of Psychology, University of Düsseldorf, Düsseldorf, Germany; 3 Department for Neurodegenerative Diseases and Geriatric Psychiatry, University Hospital Bonn, Bonn, Germany; 4 German Center for Neurodegenerative Diseases (DZNE), Bonn, Germany

**Keywords:** Obsessive-compulsive disorder, OCD, Help-seeking, Treatment barriers, Self-help, Cognitive-behavioral therapy, CBT, Exposure and response prevention, ERP, Pharmacotherapy

## Abstract

**Background:**

Individuals with obsessive-compulsive disorder (OCD) face both personal and system-based barriers in receiving first-line treatment, i.e. cognitive behavioral therapy (CBT) with exposure and response prevention (ERP). The present study comprehensively investigated help-seeking behavior, treatment barriers and facilitators, attitudes and access to gold-standard treatment in adults with OCD in Germany. We aimed to characterize the care situation and examine the influence of clinical and sociodemographic variables on help-seeking behavior and receiving treatment.

**Methods:**

An anonymous online survey was performed in individuals with OCD who were recruited in- and outside the psychiatric healthcare system. The survey included a wide range of questions regarding help-seeking behavior, treatment barriers and facilitators, attitudes towards different treatment options and access to treatment. Sociodemographic and clinical characteristics were also collected. The final sample comprised 276 individuals with OCD.

**Results:**

The mean delay to seeking psychotherapeutic treatment was *M* = 5.15 years (*SD* = 6.88) and the mean delay to recognition of OCD was *M* = 5.58 years (*SD* = 7.16). Of those 211 who had ever received CBT, 49.5% reported that therapist-guided ERP had been performed at some point during treatment. Indicators of poor healthcare, such as longer delay to recognition or a larger number of treatments before receiving ERP were significantly associated with increased symptom severity. Moreover, a younger age was associated with a shorter delay to recognition of OCD. Taboo thoughts (60.9%) and checking (52.9%) were the most commonly reported symptom dimensions, and individuals with current taboo thoughts were significantly more likely to be treated with CBT. Educational websites were identified as the most important facilitators in recognizing OCD and providing information on effective treatment options. Lack of knowledge about treatment options was reported as the most common barrier to seeking/receiving ERP-based treatment.

**Conclusions:**

Delays to the recognition of OCD and to seeking help still exceed 5 years on average, but were reduced in younger individuals, potentially reflecting increased mental health literacy. Although our sample may not be fully representative, our results fill the gap between epidemiological surveys and previous studies in outpatients. Options for improving the care situation are discussed.

**Supplementary Information:**

The online version contains supplementary material available at 10.1186/s12888-025-06655-0.

## Background

Obsessive-compulsive disorder (OCD) is a debilitating mental disorder characterized by obsessions (unwanted intrusive thoughts, images or impulses) and/or compulsions (ritualized repetitive behaviors or mental acts). In Germany, the prevalence of OCD is estimated at 3.6%, making it the fourth most common category of psychiatric disorders after anxiety, affective and substance abuse disorders [1]. With significant reductions in quality of life [2], functional impairment [3] and an increased risk for suicide attempts and death by suicide [4–6], OCD poses a huge burden to individuals, families and society [7–9]. First-line treatment for OCD includes cognitive-behavioral therapy (CBT) with exposure and response prevention (ERP) as well as medication with selective serotonin reuptake inhibitors (SSRIs). Despite high effectiveness of this treatment [10, 11], those affected by OCD face both personal and system-based barriers in receiving adequate care. First, many individuals with OCD do not seek treatment due to shame, stigmatization or because they want to handle the problem independently [12–15]. In the large German Health Interview and Examination Survey for Adults (DEGS-1), only 17.5% of subjects with a lifetime diagnosis of OCD reported having utilized any mental health service in the past 12 months, and a mere 42.7% reported having used mental health services at any time point in their lives [16]. These are the lowest rates of all mental disorders except for substance abuse disorders [16]. For other Western populations, similar findings have been reported [17, 18]. Furthermore, even if treatment is sought, waiting times for psychotherapy are long and many clinicians do not apply exposure therapy due to lack of skills or experience with OCD [19, 20]. According to a survey at a special outpatient clinic in Germany, only 20% of individuals with OCD received standard therapy consisting of CBT and SSRIs at their first point of contact for help [21]. Overall, treatment gap rates for OCD vary between 40 and 90% [22, 23], stressing the great need for novel approaches targeting the large group of undertreated and untreated individuals with OCD [24].

To overcome treatment barriers, internet-based CBT, self-help and other low-intensity treatments have been proposed [25]. Web/app-based interventions for psychiatric disorders generally increase mental health literacy and willingness to seek help [26], which may contribute to improved recognition of OCD symptoms and a shorter delay to treatment [27]. Notably, a longer delay to treatment has been linked to greater OCD severity, psychiatric comorbidity, older age, an earlier onset of OCD symptoms and the presence of contamination/cleaning symptoms [12, 28]. Although numerous studies have examined treatment-seeking behavior in outpatients with OCD [e.g. 13, 21, 29–31], studies that include individuals outside the psychiatric healthcare system are lacking. As individuals recruited via outpatient clinics, self-help organisations and social media may differ regarding clinical characteristics and help-seeking behavior [e.g. 32], employing diverse recruitment methods and comparing recruitment groups is of particular interest. Greater inclusivity could result in more robust data to inform decisions in healthcare and treatment innovations, potentially reducing disparity in treatment provision and outcomes [33]. Moreover, with growing mental health awareness and information on psychiatric disorders available online, the rate of self-diagnoses is rising. Some of the self-diagnosed individuals may seek professional help, while others may not, with significant implications for clinicians [34]. Investigating differences between professionally-diagnosed and self-diagnosed individuals with OCD, e.g. in terms of symptom severity, could yield important new insights.

In the present study, we examined help-seeking behavior, treatment barriers and facilitators, attitudes towards different treatment options and access to gold-standard treatment in adults with OCD in Germany via an anonymous online survey. To reach both individuals in- and outside the psychiatric healthcare system, we employed different levels of recruitment. The aims of our study were twofold. First, we aimed to characterize the care situation and help-seeking behavior of individuals with OCD. Second, we aimed to examine the influence of clinical and sociodemographic variables as well as recruitment group/diagnostic status on help-seeking behavior and attitudes. Based on previous studies, we hypothesized that younger individuals would seek treatment more rapidly and that a longer delay to treatment would be associated with more severe symptoms. As promoters of mental health literacy, we assumed that relevant self-help websites would be rated as important facilitators in the recognition of OCD across recruitment groups.

## Methods

### Study design and procedure

An anonymous online survey on help-seeking behavior, treatment barriers and facilitators, attitudes towards different treatment options and access to gold-standard treatment in individuals with OCD was set up using SoSci Survey [35]. To reach individuals in- and outside the psychiatric healthcare system while ensuring a certain degree of diagnostic validity, the link to the survey was disseminated via various pathways, including the outpatient clinic for OCD at the Department of Psychiatry at the University Hospital Bonn (to include outpatients with professionally validated diagnosis), the German OCD Foundation (Deutsche Gesellschaft Zwangserkrankungen e.V.; DGZ) and the German-speaking community forum of the self-help platform *OCD Land* (to include individuals with reasonably reliable diagnosis), as well as OCD Land’s Instagram page and a large German online newspaper, ZEIT ONLINE (to reach individuals with a potentially greater distance to the healthcare system, at the risk of reduced diagnostic validity). At the specialized outpatient clinic, individuals were mainly recruited before they started an ERP-based group therapy. Potential participants were informed that the survey was voluntary, anonymous, could be ended at any time without giving reasons, and would take approximately 20 min. Inclusion criteria were age ≥ 18 years and the presence of any OCD subtype. Self-diagnosis was explicitly not stated as an exclusion criterion, allowing individuals who were not in contact with a healthcare professional to participate, as well. Written informed consent was obtained online by setting a checkmark. As compensation, all participants were given the opportunity to download a short guide to exposure therapy for OCD, which was specially designed for the study. The study was in accordance with the revised Declaration of Helsinki and approved by the local ethics committee of the University Hospital Bonn (351/23-EP). The survey was available between December 2023 and May 2024, though 86% of the data (from the final dataset, see below) were collected in the first two months. The study was preregistered at OSF before the start of data collection (10.17605/OSF.IO/E4XN7). Clinical trial number: not applicable.

### Participants

In total, there were 1,752 clicks, including duplicates, to the survey link. Of the 530 individuals that gave informed consent to participate, 251 were excluded due to the following reasons: 220 only looked at first page of the survey without submitting any data, 30 terminated the survey before completing at least 80% and one participant reported an age < 18 years. To detect multiple submissions, the “identify duplicate cases” function in SPSS 27 was used, and data were visually inspected. Based on this approach, three datasets were excluded, as we recognized a characteristic style of writing (i.e. accusatory and offensive wording) in the open text field at the end of the survey, which we could link to the same known individual. We decided to exclude all three datasets from this individual due to inconsistent responses and strong concerns regarding the data’s validity. The remaining data were screened for implausible response patterns and response times (e.g. < 5 min), but no individual was excluded. The final sample consisted of *N* = 276 individuals with OCD (72.1% female, 26.4% male, 1.4% diverse). The mean age was *M* = 32.72 (*SD* = 9.28, range [18–65]). Sociodemographic information was missing for two participants. Based on recruitment access, three groups were defined: Group 1 (professionally validated diagnosis) included *n* = 32 individuals from a specialized outpatient clinic for OCD; Group 2 (a priori assumption of reasonably reliable diagnosis) comprised *n* = 126 individuals that were recruited via OCD Land’s community forum and the DGZ; and Group 3 (a priori assumption of potentially reduced diagnostic reliability) included *n* = 118 individuals that were recruited via OCD Land’s Instagram page and a ZEIT ONLINE article, or reported other access routes such as having found the survey via Google search. Across groups, 94.6% reported living in Germany, 2.2% lived in Switzerland, 1.8% lived in Austria, 0.7% lived in Luxembourg and 0.4% lived in Italy. Sociodemographic sample characteristics of the full sample and the three recruitment groups are presented in Table 1.

**Table 1:** Sociodemographic characteristics of individuals with obsessive-compulsive disorder (OCD)

	Full sample	Group 1	Group 2	Group 3	Statistic	*p*
*N*	276	32	126	118		
Age (*M*, *SD*, range)	32.72 (9.28) [18–65]	32.84 (9.84) [19–57]	32.79 (9.62) [18–65]	32.61 (8.82) [18–62]	*F*(2,272) = 0.01	0.99
Gender (*n*, %)					Χ²(4) = 1.30	0.86
Male	73 (26.4%)	9 (28.1%)	36 (28.6%)	28 (23.7%)		
Female	199 (72.1%)	23 (71.9%)	88 (69.8%)	88 (74.6%)		
Diverse	4 (1.4%)	0 (0%)	2 (1.6%)	2 (1.7%)		
Education (%)					Χ²(6) = 7.98	0.24
University degree	45.5%	40.6%	41.6%	50.8%		
A-levels (Abitur)	34.2%	43.8%	39.2%	26.3%		
Intermediate	17.8%	15.6%	17.6%	18.6%		
Lower	2.5%	0%	1.6%	4.2%		
Relationship status (%)					Χ²(2) = 1.64	0.44
Single	31.3%	40.6%	31.2%	28.8%		
In a relationship/married	68.7%	59.4%	68.8%	71.2%		
Living situation (%)					Χ²(10) = 10.51	0.40
Alone	21.1%	31.3%	22.4%	16.9%		
With partner	38.2%	25.0%	41.6%	38.1%		
With own family	21.8%	18.8%	17.6%	27.1%		
Shared apartment	8.7%	15.6%	7.2%	8.5%		
With parents/origin family	9.8%	9.4%	10.4%	9.3%		
Assisted living	0.4%	0%	0%	0.4%		
Working status (%)					Χ²(10) = 18.31	0.050
Full	44.4%	31.3%	38.4%	54.2%		
Part-time	24.0%	18.8%	25.6%	23.7%		
Student	16.4%	28.1%	17.6%	11.9%		
Housewife/husband	2.9%	9.4%	2.4%	1.7%		
No current job	7.3%	9.4%	8.8%	5.1%		
Disability pension	5.1%	3.1%	7.2%	3.4%		
Urbanicity (%)					Χ²(8) = 8.79	0.36
> 1 Mio.	15.6%	15.6%	12.8%	18.6%		
500.000–1 Mio.	9.1%	9.4%	11.2%	6.8%		
100.000–500.000	21.1%	34.4%	20.0%	18.6%		
10.000–100.000	28.0%	28.1%	26.4%	29.7%		
< 10.000	26.2%	12.5%	29.6%	26.3%		
Socioeconomic status (*M*, *SD*, range)*	6.28 (2.13) [1–10]	6.06 (2.09) [2–10]	6.29 (2.18) [1–10]	6.33 (2.09) [1–10]	*F*(2,272) = 0.20	0.82

Group 1 was recruited via a specialized outpatient unit for OCD; Group 2 was recruited via OCD Land’s community forum and the DGZ; and Group 3 was recruited via broader access routes such as OCD Land’s Instagram page and an article in a German online newspaper

* Socioeconomic status was assessed on a subjective scale from 1 to 10

### Measures

We employed a wide range of questions regarding help-seeking behavior (e.g. “Have you ever sought psychotherapeutic treatment for your OCD?”; response format: yes/no), treatment barriers and facilitators (e.g. “How many therapists have you contacted in the course of your search for treatment?”; response format: number), attitudes towards different treatment options (e.g. “If there were no barriers in the healthcare system, which treatment setting would you use?”; rating different options on a 5-point Likert scale ranging from 1 (in no case) to 5 (in any case): outpatient individual therapy, outpatient group therapy, self-help literature…) and access to ERP treatment (e.g. “What influence did the following factors have on the fact that you have not sought/received exposure-based treatment to date or in the past?”; rating the contribution of different barriers on a 5-point Likert scale ranging from 1 (none) to 5 (very large): lack of knowledge about treatment options and contact points, waiting lists for therapists being too long, treatment services too far away…). All healthcare-related questions and response options were developed in consultation with individuals affected by OCD, and the full list can be found in the Supplement. Sociodemographic and clinical characteristics were also collected, including age at first obsessive-compulsive symptoms (“How old were you when you first started experiencing obsessive-compulsive symptoms?”), age at OCD onset (“How old were you at the beginning of your OCD, i.e. when the obsessive-compulsive symptoms reached such a high level that you felt impaired or suffered from them?”), comorbidities (both professionally diagnosed and self-diagnosed) and quality of life (“On a scale from 1 to 10, how would you rate your overall quality of life (with respect to well-being, social relationships, health, participation in life…)?”).

The severity of OCD symptoms was measured using a self-rated version of the *Yale-Brown Obsessive-Compulsive Scale* (Y-BOCS) [36, 37]. Medium-to-strong correlations of Y-BOCS interview and self-rated scores have been reported, with patients tending to rate symptoms lower than clinicians [38–41]. The Y-BOCS comprises ten items that are rated on a 5-point Likert scale ranging from 0 to 4, plus an additional six items assessing OCD-related characteristics (insight, avoidance, decision-making difficulties, inflated responsibility, slowness, pathological doubting) that are not included in the total score. Due to a programming error, item 9 of the Y-BOCS was missing for all participants. Considering that for the resistance items (4 and 9), low item-remainder correlations, low correlations across scale versions (interview vs. self-rated) and low sensitivity to symptom change have consistently been reported [38–44], we decided to employ an alternative computation method suggested by Woody et al. [44]. Accordingly, the Y-BOCS total score was calculated excluding items 4 and 9, but including the avoidance item instead. In Table 2, we also report a second Y-BOCS total score based on the original items, with the missing item’s value set equal to the mean value of all other items for each subject, in order to increase comparability with other studies.

**Table 2:** *Clinical characteristics of individuals with obsessive-compulsive disorder (OCD).*

	Full sample	Group 1	Group 2	Group 3	Statistic	*p*
Y-BOCS (*M*, *SD*, range)						
Y-BOCS (Woody et al. 1995) ^†^	19.71 (5.17) [8–30]	20.56 (5.14) [10–29]	19.65 (4.75) [8–30]	19.54 (5.61) [8–30]	*F*(2,273) = 0.50	0.60
Y-BOCS (compromised) ^‡^	22.59 (4.99) [8.89–33.3]	23.19 (5.60) [11.1–32.2]	22.59 (4.60) [8.89–33.3]	22.34 (5.25) [8.89–33.3]	*F*(2,273) = 0.29	0.75
Age at onset (*M*, *SD*, range)						
OC symptoms	16.92 (8.97) [3–53]	13.56 (7.36) [3–35]	17.44 (9.11) [4–45]	17.29 (9.08) [3–53]	*F*(2,273) = 2.58	0.078
OCD	21.91 (8.77) [7–56]	20.72 (9.66) [7–50]	21.98 (8.88) [7–56]	22.16 (8.45) [7–55]	*F*(2,273) = 0.35	0.71
OCD symptom dimensions (%)						
Washing/contamination					Χ²(4) = 11.11	0.025
Currently	28.3%	53.1%	24.6%	25.4%		
In the past	20.7%	12.5%	22.2%	21.2%		
Never	51.1%	34.4%	53.2%	53.4%		
Checking					Χ²(4) = 14.36	0.006
Currently	52.9%	62.5%	42.1%	61.9%		
In the past	21.0%	6.3%	27.0%	18.6%		
Never	26.1%	31.3%	31.0%	19.5%		
Order/symmetry					Χ²(4) = 2.62	0.62
Currently	13.8%	15.6%	14.3%	12.7%		
In the past	16.3%	25.0%	15.9%	14.4%		
Never	69.9%	59.4%	69.8%	72.9%		
Taboo thoughts					Χ²(4) = 3.22	0.52
Currently	60.9%	50.0%	65.9%	58.5%		
In the past	14.9%	18.8%	12.7%	16.1%		
Never	24.3%	31.3%	21.4%	25.4%		
Magical thinking/repeating					Χ²(4) = 4.30	0.37
Currently	33.3%	31.3%	27.8%	39.8%		
In the past	25.7%	25.0%	27.0%	24.6%		
Never	40.9%	43.8%	45.2%	35.6%		
Somatic obsessions/compulsions					Χ²(4) = 9.43	0.051
Currently	30.4%	18.8%	27.0%	37.3%		
In the past	15.2%	6.3%	17.5%	15.3%		
Never	54.3%	75.0%	55.6%	47.5%		
Comorbidities (%)						
Depression					Χ²(4) = 3.63	0.46
Professional diagnosis	45.3%	46.9%	46.8%	43.2%		
Self-diagnosed	9.8%	9.4%	6.3%	13.6%		
Anxiety disorder					Χ²(4) = 6.48	0.17
Professional diagnosis	38.8%	31.3%	38.1%	41.5%		
Self-diagnosed	19.2%	12.5%	16.7%	23.7%		
PTSD					Fisher’s exact test = 9.55	0.037
Professional diagnosis	8.0%	3.1%	4.8%	12.7%		
Self-diagnosed	6.5%	12.5%	4.0%	7.6%		
OCD-related disorder					Fisher’s exact test = 4.86	0.29
Professional diagnosis	6.5%	3.1%	8.7%	5.1%		
Self-diagnosed	11.6%	21.9%	10.3%	10.2%		
Anorexia					-	
Professional diagnosis	3.3%	0%	4.0%	3.4%		
Self-diagnosed	0.7%	0%	1.6%	0%		
ADHD					Fisher’s exact test = 17.18	0.001
Professional diagnosis	6.5%	18.8%	7.9%	1.7%		
Self-diagnosed	6.5%	3.1%	3.2%	11.0%		
Autism spectrum disorder					-	
Professional diagnosis	0.4%	3.1%	0%	0%		
Self-diagnosed	3.3%	3.1%	3.2%	3.4%		
PHQ-9 (*M*, *SD*, range)	12.59 (6.12) [0–27]	13.31 (6.68) [1–25]	12.14 (5.80) [1–27]	12.88 (6.30) [0–27]	*F*(2,273) = 0.69	0.50
Acceptance scale (*M*, *SD*, range)						
Sum score	38.73 (11.85) [15–72]	35.47 (10.95) [17–57]	38.49 (12.51) [15–72]	39.87 (10.85) [16–67]	*F*(2,273) = 1.85	0.16
Non-judging subscale	22.76 (8.34) [8–40]	21.53 (9.09) [8–40]	22.57 (8.55) [8–40]	23.30 (7.92) [9–40]	*F*(2,273) = 0.62	0.54
Non-reactivity subscale	15.97 (5.49) [7–34]	13.94 (5.16) [7–29]	15.92 (5.46) [7–34]	16.58 (5.52) [7–32]	*F*(2,273) = 2.96	0.054
Quality of life (*M*, *SD*, range)*	5.97 (2.11) [1–10]	5.56 (2.23) [1–10]	5.91 (2.07) [1–10]	6.14 (2.12) [1–10]	*F*(2,272) = 1.00	0.37

Group 1 was recruited via a specialized outpatient unit for OCD; Group 2 was recruited via OCD Land’s community forum and the DGZ; and Group 3 was recruited via broader access routes such as OCD Land’s Instagram page and an article in a German online newspaper

ADHD, Attention deficit hyperactivity disorder; OC, obsessive-compulsive; OCD, obsessive-compulsive disorder; *SD*, standard deviation; PHQ-9, Patient Health Questionnaire 9; PTSD, post-traumatic stress disorder; Y-BOCS, Yale-Brown Obsessive Compulsive Scale, self-rated version

^†^ In accordance with Woody et al. (1995), the Y-BOCS total score was calculated excluding items 4 and 9 (the latter was missing in all datasets due to a programming error), but including the avoidance item instead. Thus, this sum score was based on nine items

^‡^ In order to increase comparability with other studies, we also report a compromised Y-BOCS total score based on the original items, with the missing item’s value (i.e. items 9) set equal to the mean value of all other items for each subject

* Quality of life was assessed on a subjective scale from 1 to 10

Depressive symptom severity was measured via the *Patient Health Questionnaire-9* (PHQ-9) [45, 46]. The PHQ‐9 is a well-established self-rating scale comprising nine items that are rated on a 4‐point Likert scale ranging from 0 (not at all) to 3 (almost every day).

To assess the acceptance of emotions and thoughts, the “non-judging” (8 items) and “non-reactivity” (7 items) subscales of the Five Facet Mindfulness Questionnaire (FFMQ-D) [47] were employed. In accordance with previous studies, these two subscales are referred to as the *Acceptance Scale* (AS) [48]. Items were rated on a 5-point Likert scale ranging from 1 (never or very rarely true) to 5 (very often or always true). Acceptance of negative internal experiences is associated with improved psychological health [49] and can thus be seen as an indicator of reduced OCD symptom severity.

### Statistical analyses

Data were analyzed using IBM SPSS 27. For continuous measures, group differences were assessed using ANOVAs (i.e. comparing the three recruitment groups) or *t*-tests (e.g. self-diagnosis vs. professional diagnosis). For categorical variables, we employed chi-square tests or Fisher’s exact tests if expected values were low. To analyze the influence of sociodemographic variables on the receipt of CBT in general and CBT with ERP, binary logistic regressions were conducted. Pearson correlations were computed to investigate associations between continuous measures, i.e. age at first obsessive-compulsive symptoms, age at OCD onset, obsessive-compulsive (Y-BOCS) and depressive (PHQ-9) symptom severity, acceptance (AS) and quality of live. Spearman correlations were conducted for variables that were not normally distributed, i.e. delay to recognition of the symptoms as OCD, delay to seeking professional help, number of contacted professionals and number of treatments before receiving ERP. Sex effects were also examined via *t*-tests and chi-square tests, excluding the four individuals who identified as diverse, due to their small number. Given the explorative nature of the study and substantial inter-correlations of many measures, a *p*-value < 0.05 was considered significant.

## **Results**

### Clinical characterization and differences between recruitment groups

The three recruitment groups did not differ significantly with respect to age, gender, Y-BOCS total score, age at first obsessive-compulsive symptoms and age at OCD onset, PHQ-9, AS and quality of life (all *p* > 0.05; see Tables 1 and 2).

Overall, taboo thoughts (60.9%, currently, 14.9% in the past) and checking (52.9% currently, 21.0% in the past) were the most commonly reported symptom dimensions, followed by magical thinking/repeating (33.3% currently, 25.7% in the past), somatic obsessions/compulsions, e.g. hyperawareness OCD (also known as sensorimotor OCD; 30.4% currently, 15.2% in the past), and washing/contamination symptoms (28.3% currently, 20.7% in the past). Order/symmetry symptoms were only reported by a few participants (13.8% currently, 16.3% in the past). The three recruitment groups differed only regarding the presence of washing/contamination (Χ²(4) = 11.11, *p* = 0.025, Cramer-*V* = 0.14) and checking symptoms (Χ²(4) = 14.36, *p* = 0.006, Cramer-*V* = 0.16), with current washing/contamination symptoms being more common in Group 1. Current checking symptoms were less common in Group 2, while past checking symptoms were more common, suggesting that a larger proportion of individuals in this group had experienced a shift from checking to other OCD symptoms. The proportion of individuals who had never experienced checking symptoms was also smaller in Group 3.

With respect to comorbidities, depressive (45.3% professionally diagnosed, 9.8% self-diagnosed) and anxiety disorders (38.8% professionally diagnosed, 19.2% self-diagnosed) were the most common, followed by post-traumatic stress disorder (PTSD; 8.0% professionally diagnosed, 6.5% self-diagnosed), OCD-related disorders (6.5% professionally diagnosed, 11.6% self-diagnosed) and attention deficit hyperactivity disorder (ADHD; 6.5% professionally diagnosed, 6.5% self-diagnosed). Anorexia (3.3% professionally diagnosed, 0.7% self-diagnosed) and autism-spectrum disorder (0.4% professionally diagnosed, 3.3% self-diagnosed) were only reported by a few participants. The three recruitment groups differed only with respect to the presence of PTSD (Fisher’s exact test = 9.55, *p* = 0.037, Cramer-*V* = 0.14) and ADHD (Fisher’s exact test = 17.18, *p* = 0.001, Cramer-*V* = 0.18). While professional PTSD diagnoses were overrepresented in Group 3, self-diagnosed PTSD was more common in Group 1. Conversely, self-diagnosed ADHD was more common in Group 3 and professional ADHD diagnoses were overrepresented in Group 1, presumably because the Department of Psychiatry and Psychotherapy at the University Hospital Bonn has a strong focus on adult ADHD research and treatment.

### Comparing professionally-diagnosed and self-diagnosed individuals with OCD

Overall, 80.1% of participants reported that they had received their OCD diagnosis from a healthcare professional, while 19.9% endorsed self-diagnosis. Expectedly, the proportion of individuals with self-diagnosis was larger in Group 3 than in Group 2 (25.4% vs. 15.9%), but this difference was not statistically significant (Χ²(1) = 3.41, *p* = 0.065, Cramer-*V* = 0.12). Participants with current or past taboo thoughts reported significantly more professional diagnoses than self-diagnoses (Χ²(2) = 7.56, *p* = 0.023, Cramer-*V* = 0.17), while no group differences were observed for other symptom dimensions (all *p* > 0.05). Individuals with a professional diagnosis reported a significantly younger age at first obsessive-compulsive symptoms (*t*(67.68) = 2.21, *p* = 0.031, *d* = 0.41), a younger age at OCD onset (*t*(65.72) = 2.36, *p* = 0.021, *d* = 0.46), and a longer duration of OCD (*t*(273) = -2.20, *p* = 0.029, *d* = -0.33). Notably, there were no significant associations of diagnostic status (professional vs. self-diagnosis) with age, gender, duration of obsessive-compulsive symptoms, Y-BOCS total score, PHQ-9, AS, and quality of life (all *p* > 0.05). In addition, participants with verified professional diagnosis (Group 1) and participants with self-reported professional diagnosis did not differ with regard to these measures (all *p* > 0.05).

### Description of the care situation

In our sample, 88.4% of participants reported that they had sought psychotherapeutic help for treating their OCD. While the mean delay to seeking (not necessarily receiving) psychotherapeutic help was *M* = 5.15 years (*SD* = 6.88), the mean delay to recognition of the symptoms as OCD was *M* = 5.58 years (*SD* = 7.16). Of those who had ever sought professional help, 38.7% reported that they had recognized their symptoms as OCD on their own before seeking help, 30.7% reported that delay to recognition and delay to seeking help were identical, and another 30.7% reported that recognition of their symptoms as OCD happened later than their first search for professional help. Furthermore, 52.7% of individuals with self-diagnosed OCD reported that they had ever sought professional help.

The median number of professionals contacted before receiving psychotherapy was *Md* = 5 (*M* = 8.94, *SD* = 11.99, range [1–99]). With respect to outpatient treatment, 74.3% of individuals reported that they had ever (currently or in the past) received CBT, 32.2% had ever received psychodynamic psychotherapy, 12.3% had ever received psychoanalysis and 7.6% had ever received systemic therapy. With respect to inpatient or day-care treatment, 31.2% of individuals had ever received CBT-based treatment, 10.1% had ever received psychodynamic-based treatment and 15.6% reported that they had ever received other forms of inpatient or day-care treatment. Of those who had ever received CBT in either setting, 49.5% reported that therapist-guided ERP had been performed at some point during treatment and 71.2% reported that ERP had been assigned as homework. The median number of therapies started until receiving ERP was *Md* = 2 (*M* = 2.64, *SD* = 2.63, range [1–20]).

With regard to psychopharmacological medication, 40.6% of individuals reported that they were currently medicated with SSRIs, 21.0% had been medicated with SSRIs in the past, 36.6% had never been medicated with SSRIs, and 1.8% were not sure. 6.9% of individuals reported that they were currently medicated with SSRIs augmented by an antipsychotic drug, 15.6% had been medicated with SSRIs plus antipsychotics in the past, 73.9% had never been medicated with SSRIs plus antipsychotics, and 3.6% were not sure. Only 1.1% of individuals reported that they were currently medicated with clomipramine, 2.5% had been medicated with clomipramine in the past, 92.0% had never been medicated with clomipramine, and 4.3% were not sure.

### Clinical and sociodemographic characteristics associated with the receipt of CBT

Across groups, *n* = 121 participants reported that they were currently treated with CBT as in- or outpatients, whereas *n* = 155 were not. Individuals currently receiving CBT were significantly younger (*t*(272.73) = -2.07, *p* = 0.040, *d* = -0.24), had a shorter duration of obsessive-compulsive symptoms (*t*(273) = -2.65, *p* = 0.009, *d* = -0.32), a shorter duration of OCD (*t*(272) = -2.18, *p* = 0.030, *d* = -0.24), and a higher quality of life (*t*(273) = 2.34, *p* = 0.018, *d* = 0.29). Furthermore, individuals with current taboo thoughts were significantly more likely to be currently treated with CBT (Χ²(1) = 7.96, *p* = 0.005, Cramer-*V* = 0.17), whereas there were no significant associations with other current symptom dimensions (all *p* > 0.05). With regard to comorbidities, there was a significant negative effect of comorbid anxiety disorders (Χ²(2) = 11.63, *p* = 0.003, Cramer-*V* = 0.21), indicating that individuals without this comorbidity were more likely to currently receive CBT, and individuals with a self-diagnosed anxiety disorder were less likely. There were no significant associations with other comorbidities, age at first obsessive-compulsive symptoms, age at OCD onset, delay to recognition of OCD, delay to seeking professional help, Y-BOCS total score, PHQ-9, AS, and gender (all *p* > 0.05). Notably, recruitment groups did also not differ regarding the current receipt of CBT (*p* > 0.05).

A binary regression to predict the receipt of current CBT based on sociodemographic variables (age, gender, education, relationship status, socioeconomic status, and living situation) did not yield a significant fit of the overall model (*p* > 0.05), despite a significant contribution of age (see above).

### Clinical and sociodemographic characteristics associated with the receipt of CBT with ERP

Next, we compared individuals that had ever received CBT with ERP (either therapist-guided or as homework; *n* = 159) to those who had only ever received CBT without ERP (*n* = 52). Individuals with OCD who had ever received CBT with ERP reported a significantly younger age at first obsessive-compulsive symptoms (*t*(209) = -2.35, *p* = 0.020, *d* = -0.38), a younger age at OCD onset (*t*(209) = -2.13, *p* = 0.034, *d* = -0.34), a lower level of depressive symptoms (*t*(209) = -2.12, *p* = 0.035, *d* = -0.34), and a longer delay to seeking professional help (Mann-Whitney-*U* = 4,863, *p* = 0.005, *r* = 0.20). Moreover, individuals who had never experienced any washing/contamination symptoms (currently or in the past) were significantly less likely to have ever received ERP during CBT (Χ²(1) = 7.18, *p* = 0.007, Cramer-*V* = 0.18). The presence of all other symptom dimensions (currently or in the past vs. never) was not significantly related to the receipt of CBT with ERP (all *p* > 0.05). With respect to comorbidities, there was a significant negative association between the receipt of ERP and comorbid anxiety disorders (Χ²(2) = 7.58, *p* = 0.023, Cramer-*V* = 0.19; similar to Sect. 3.4). Specifically, individuals with a self-diagnosed anxiety disorder were less likely to have ever received ERP, while individuals without any comorbid anxiety disorder were more likely. We did not observe any significant associations with other comorbidities, delay to recognition, duration of obsessive-compulsive symptoms or OCD, Y-BOCS total score, AS, quality of life, age, and gender (all *p* > 0.05). Recruitment groups did also not differ regarding the receipt of CBT with ERP (*p* > 0.05).

A binary regression to predict the receipt of ERP based on sociodemographic variables (age, gender, education, relationship status, socioeconomic status, and living situation) did not yield any significant effects (all *p* > 0.05).

### Characteristics of individuals who have never sought psychotherapy

In the full sample, *n* = 32 individuals reported that they had never sought any psychotherapeutic treatment. These individuals were more likely to be male (Χ²(1) = 6.75, *p* = 0.009, Cramer-*V* = 0.16) and to have current order/symmetry symptoms (Fisher’s exact test = 7.84, *p* = 0.017, Cramer-*V* = 0.18), while they were less likely to have current taboo thoughts (Fisher’s exact test = 8.52, *p* = 0.014, Cramer-*V* = 0.17). With respect to the other symptom dimension, there were no significant group differences. Moreover, there were no significant associations with age, delay to recognition of OCD, duration of obsessive-compulsive symptoms or OCD, Y-BOCS total score, PHQ-9, AS, and quality of life (all *p* > 0.05). Expectedly, all of these individuals were in Group 3.

### Assessment of treatment barriers and facilitators

**Fig. 1:**
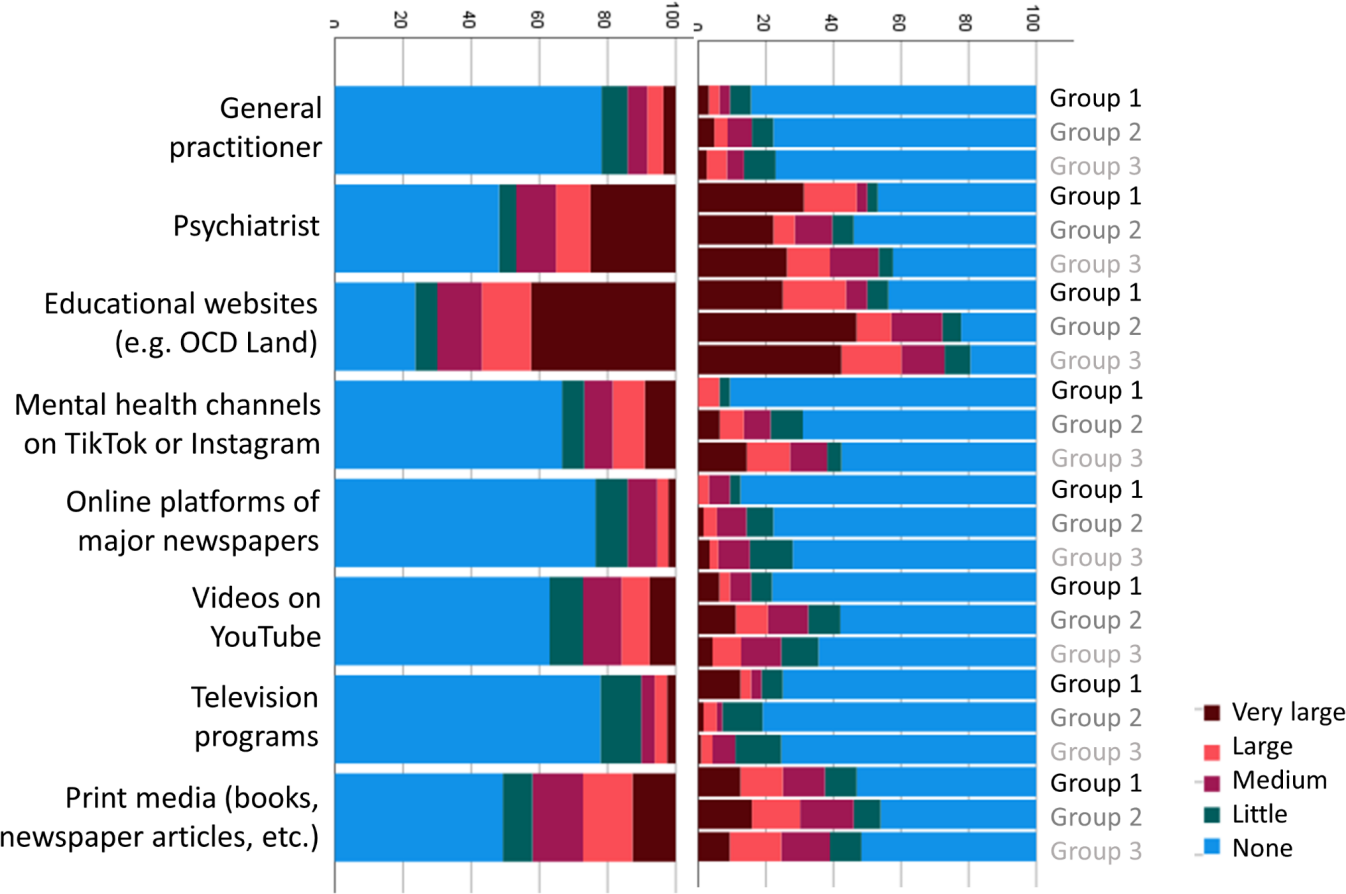
Subjective contribution of different facilitators in recognizing OCD

**Fig. 2:**
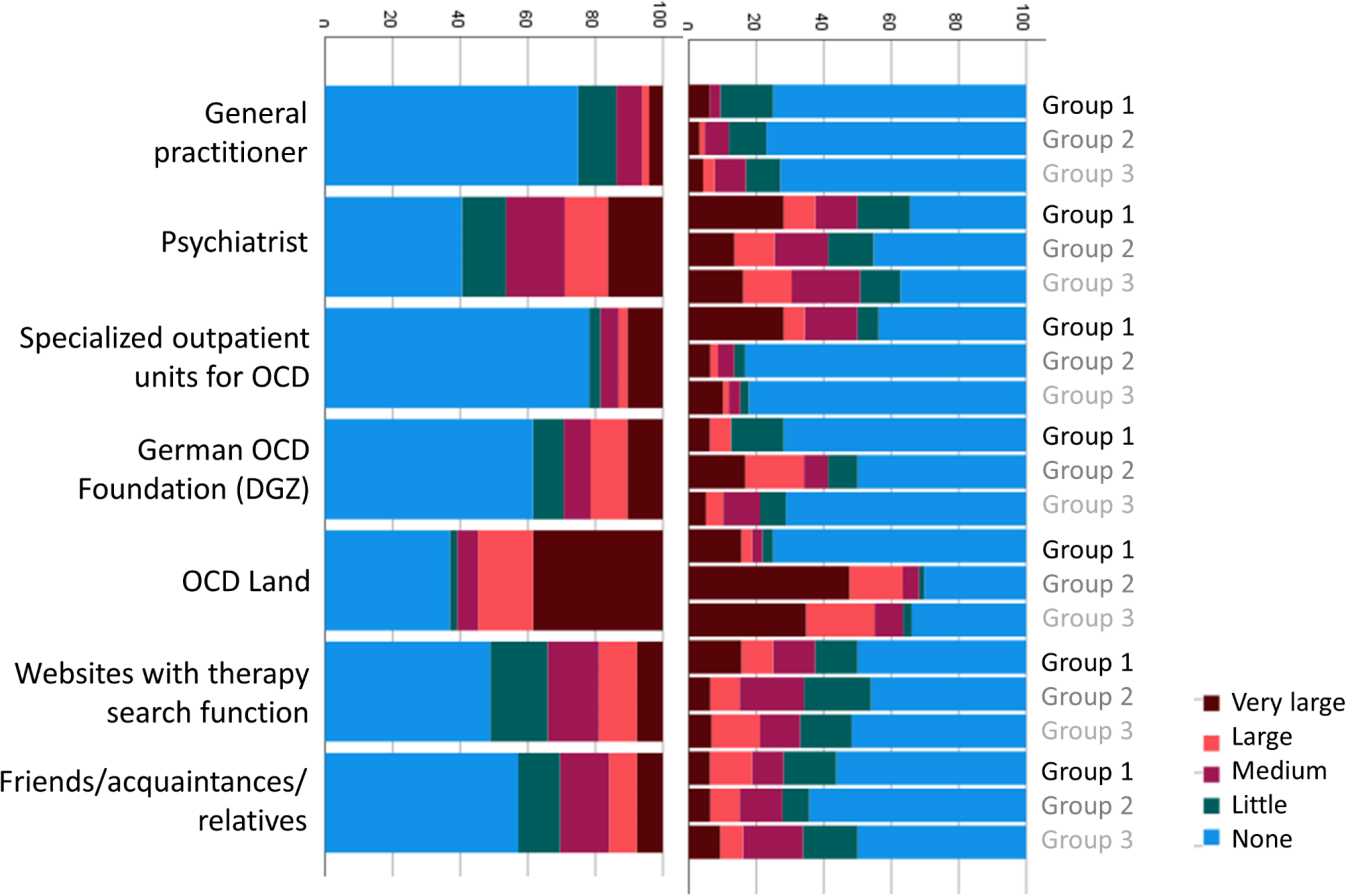
Subjective contribution of different facilitators in obtaining information about effective treatment options for OCD

**Fig. 3:**
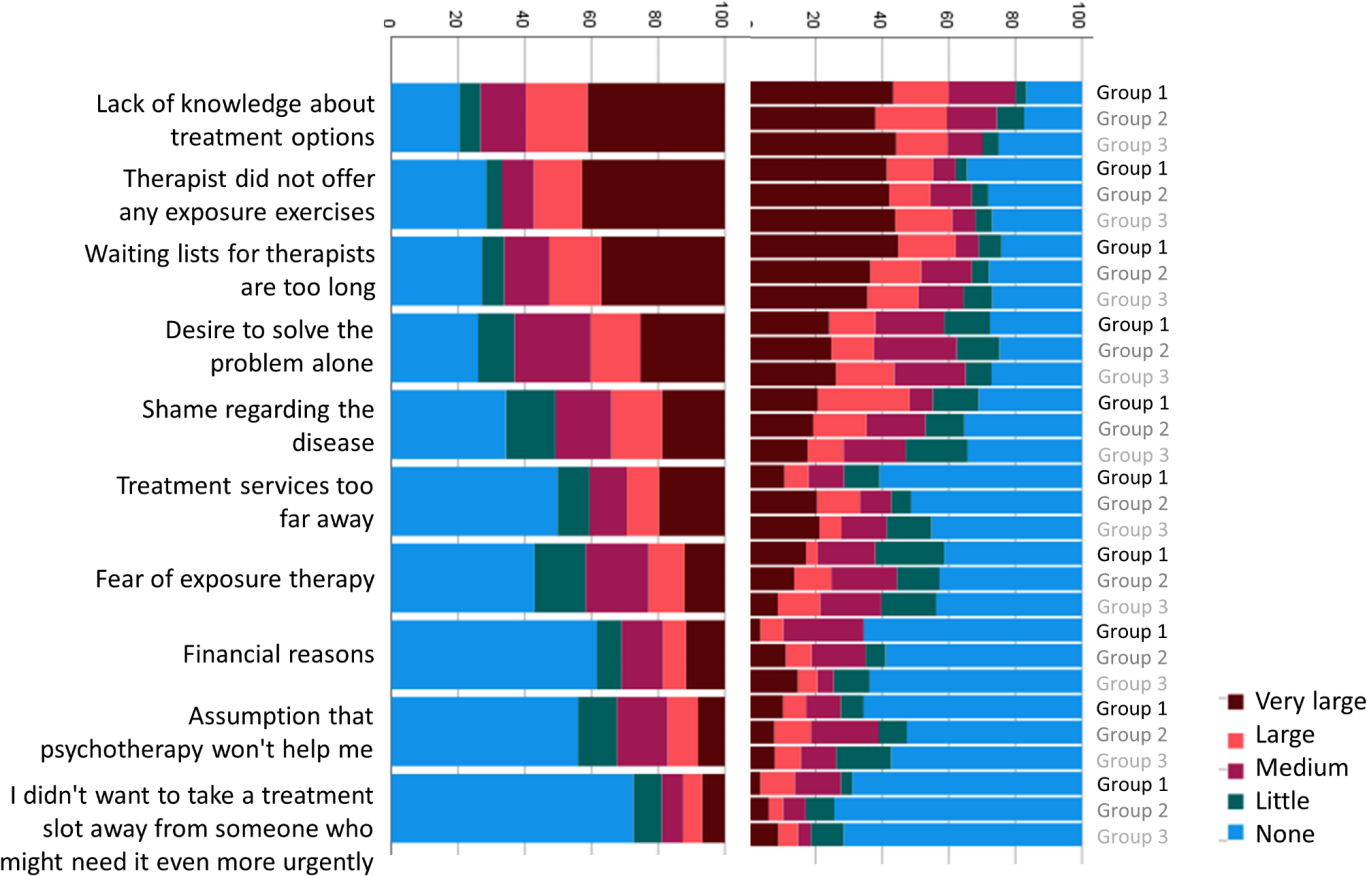
Subjective contribution of different barriers to seeking/receiving exposure-based treatment to date or in the past

**Fig. 4:**
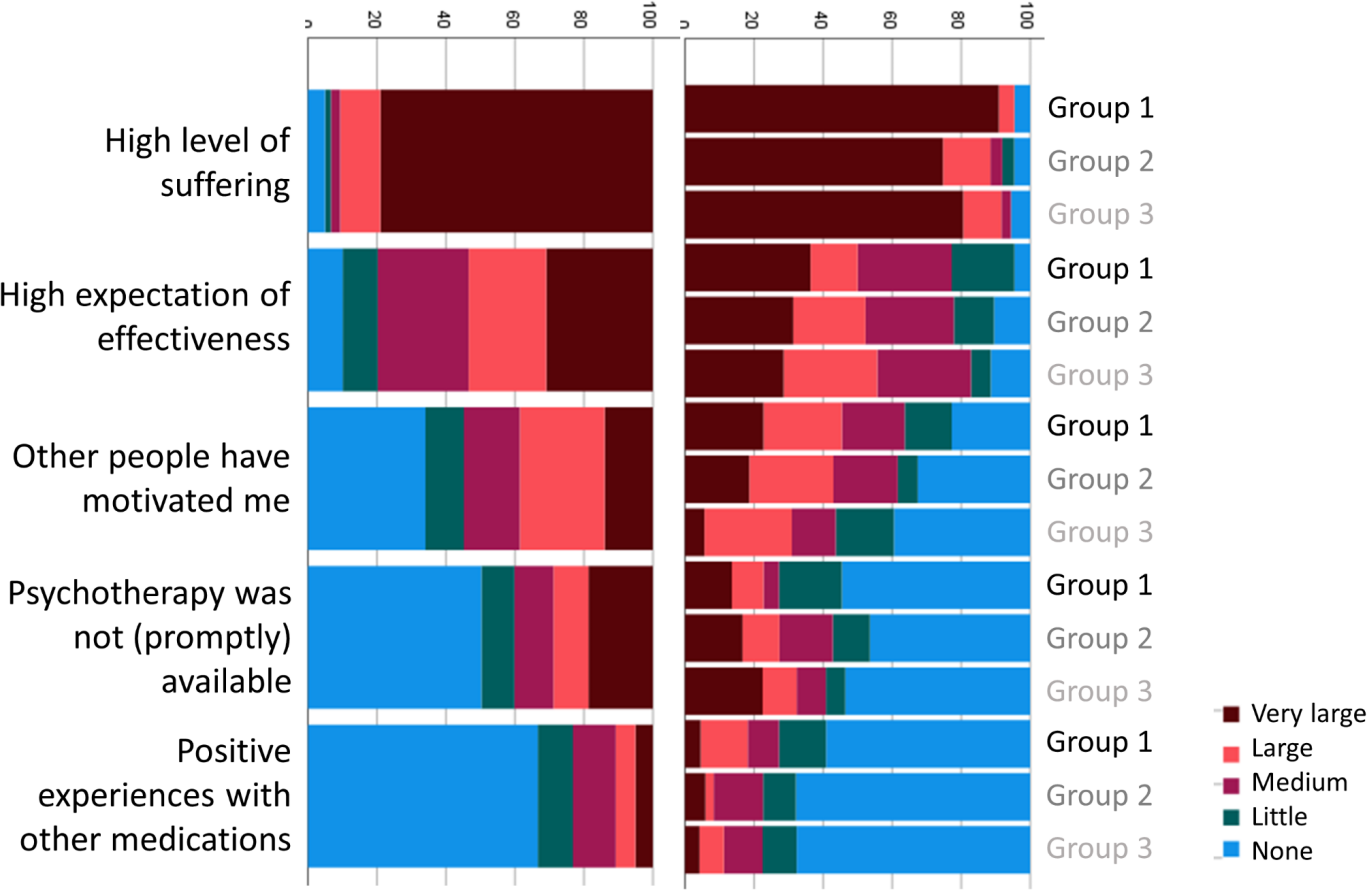
Subjective contribution of different facilitators of medical treatment for OCD

**Fig. 5:**
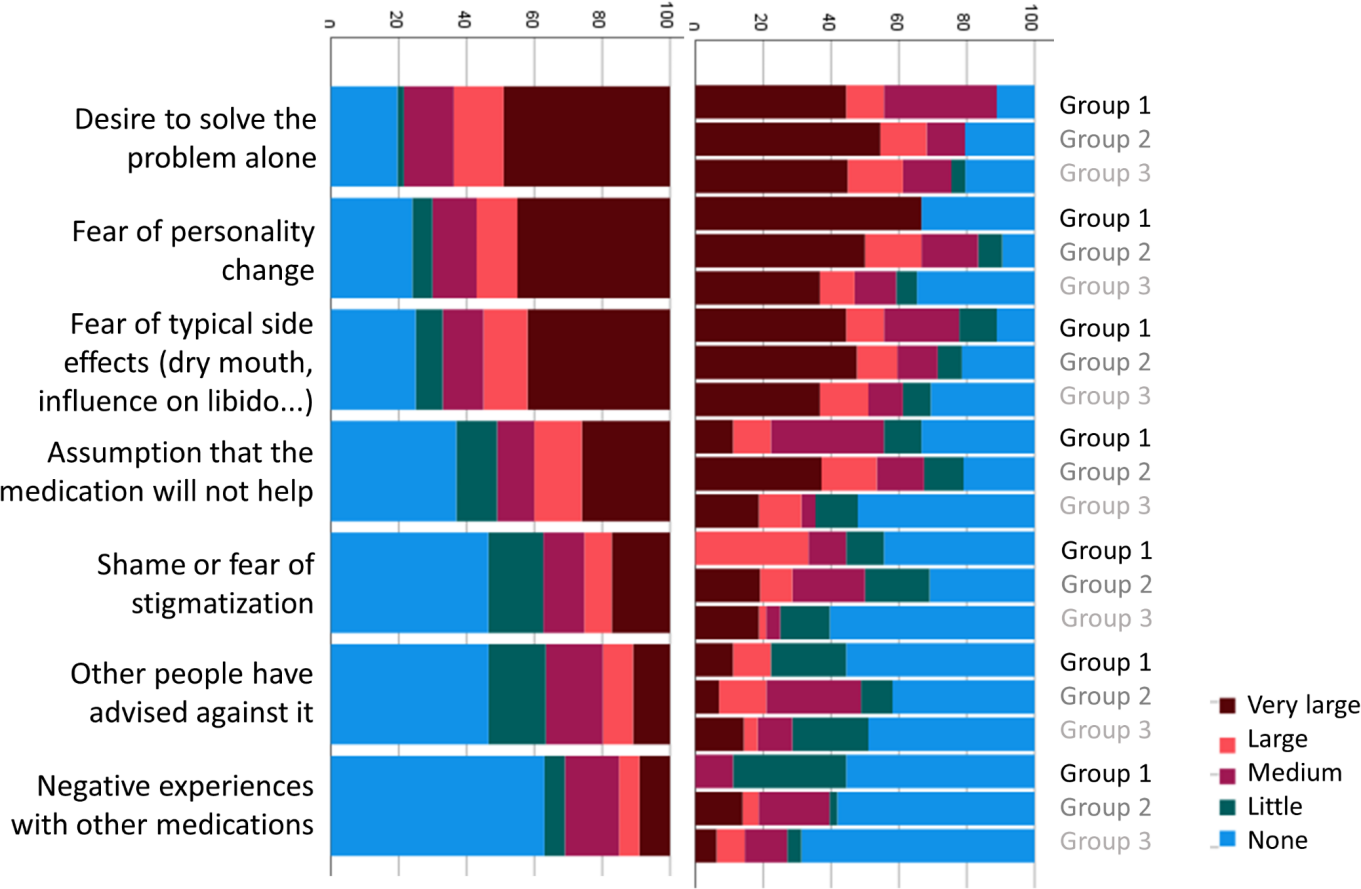
Subjective contribution of different barriers to medical treatment for OCD

Participants were asked to rate the contribution of different contact points and media for the recognition of their symptoms as OCD, and for obtaining information about effective treatment options for OCD. Responses are depicted in Figs. 1 and 2, both separated by group and for the full sample. Next, participants rated the influence of different barriers to receiving ERP (Fig. 3). Finally, participants were asked to rate the impact of different factors on their decision to either take medication for their OCD or to decline medical treatment (Figs. 4 and 5).

### Attitudes towards different treatment options

**Fig. 6:**
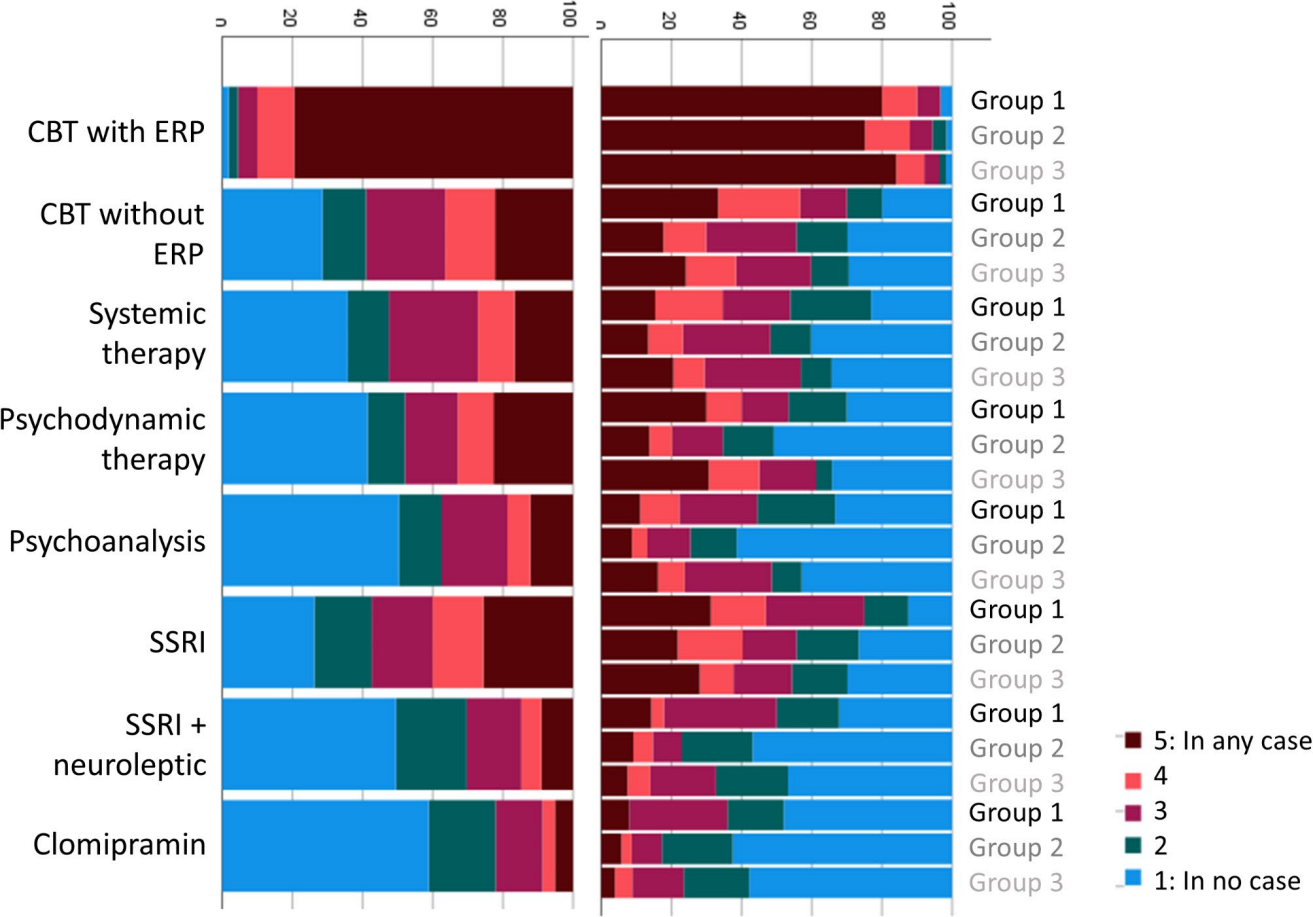
Preference of treatment options if there were no external barriers

**Fig. 7:**
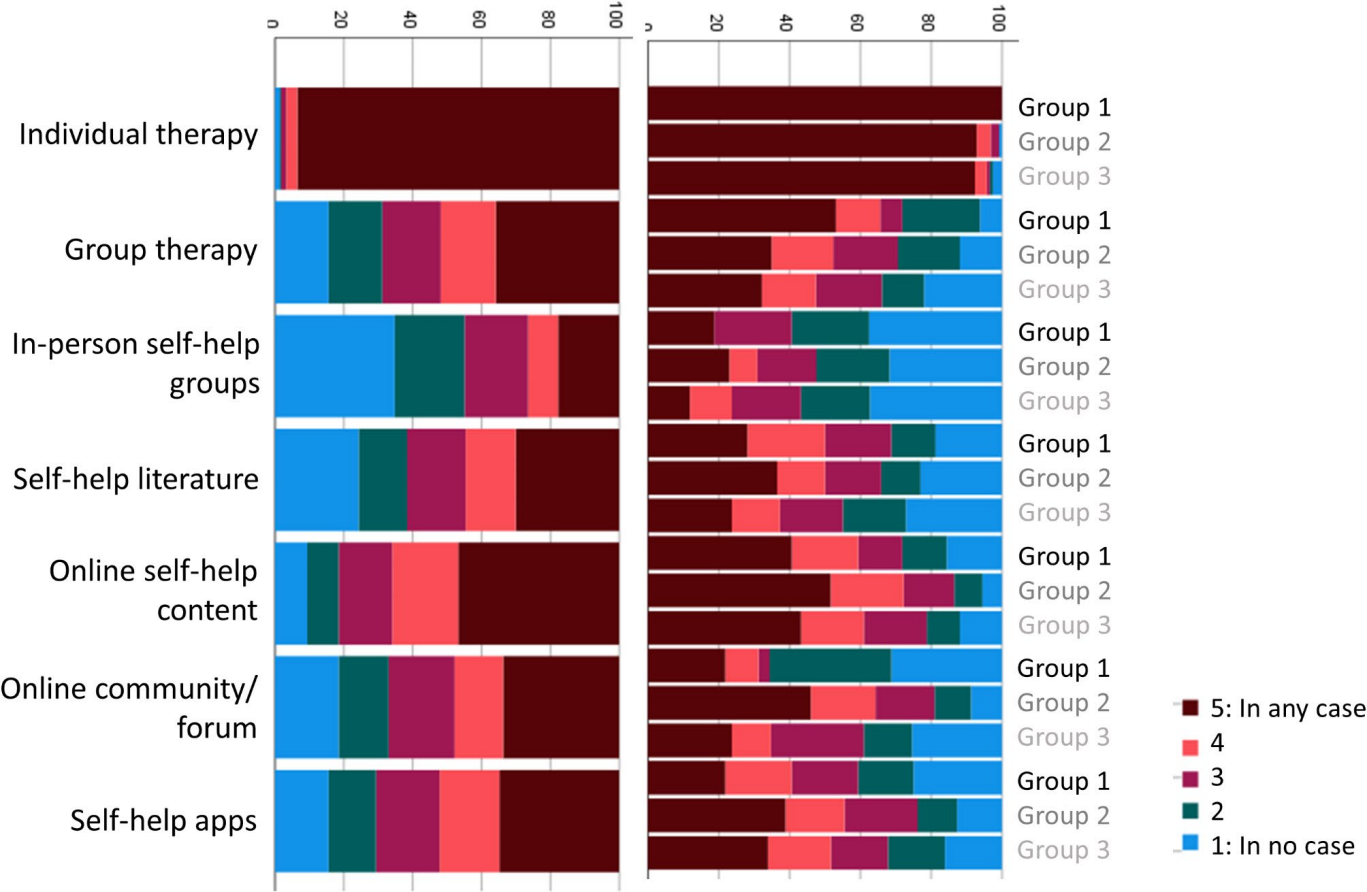
Preference of treatment settings if there were no external barriers

Participants were then asked to rate which treatment options and which treatment settings they would use to treat their OCD if there were no barriers in the healthcare system. Responses are depicted in Figs. 6 and 7.

### Usage of self-help resources

**Fig. 8:**
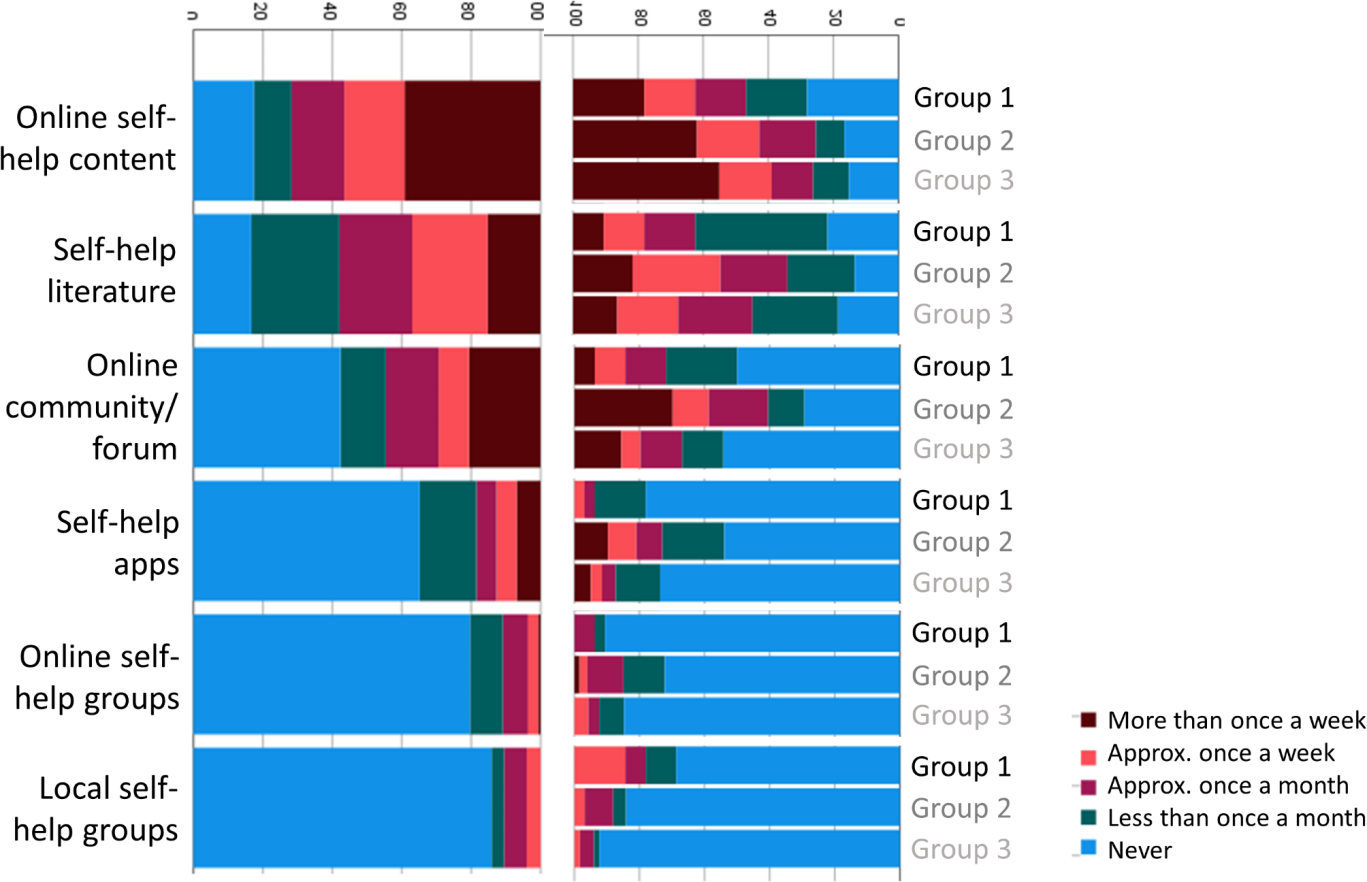
Usage of different self-help resources

Self-help resources were used by the majority of participants, with online self-help content and self-help literature being the most popular (Fig. 8). Across all categories of self-help resources, Group 1 reported the lowest usage rate. Although individuals from this group were the least likely to use online resource, over a third endorsed using online self-help content at least once a week.

### Correlations

The Y-BOCS total score was significantly associated with age at first obsessive-compulsive symptoms (*r* = -0.19, *p* = 0.002), age at OCD onset (*r* = -0.15, *p* = 0.012), delay to recognition of the symptoms as OCD (ρ = 0.20, *p* = 0.001), delay to seeking professional help (ρ = 0.15, *p* = 0.018), number of contacted professionals (ρ = 0.15, *p* = 0.022), number of treatments before receiving ERP (ρ = 0.24, *p* = 0.002), PHQ-9 (*r* = 0.64, *p* < 0.001), AS (*r* = -0.53, *p* < 0.001), and quality of life (*r* = -0.51, *p* < 0.001). There were no significant correlations with age and duration of obsessive-compulsive symptoms or OCD (*p* > 0.05).

Notably, age was significantly associated with delay to recognition of OCD (ρ = 0.20, *p* = 0.001), delay to seeking professional help (ρ = 0.19, *p* = 0.003), number of contacted professionals (ρ = -0.21, *p* = 0.002), and AS (*r* = 0.20, *p* = 0.01). There were no significant correlations of age with PHQ-9 and quality of life (*p* > 0.05).

With regard to self-reported barriers to ERP, we observed significant positive correlations of the Y-BOCS total score with being afraid of ERP (ρ = 0.23, *p* < 0.001), treatment options being too far away (ρ = 0.22, *p* < 0.001), too long waiting times for treatment (ρ = 0.14, *p* = 0.026), and assuming that psychotherapy would not work for me (ρ = 0.18, *p* = 0.005). The other barriers (see Fig. 1 for all barriers) were not significantly related to OCD symptom severity (*p* > 0.05).

Age was negatively correlated with the assumption that psychotherapy would not work for me (ρ = -0.16, *p* = 0.010). All other barriers to ERP showed no significant association with age (*p* > 0.05).

### The role of OCD Land

To examine the specific role of the self-help platform OCD Land for the healthcare situation of individuals with OCD in Germany, we compared individuals that were recruited via the community forum or Instagram page of OCD Land (*n* = 176) with all other participants (*n* = 100). Individuals recruited via OCD Land reported a significantly older age at first obsessive-compulsive symptoms (*t*(274) = 2.27, *p* = 0.024, *d* = 0.28), an older age at OCD onset (*t*(273) = 2.64, *p* = 0.009, *d* = 0.33), a shorter duration of obsessive-compulsive symptoms (*t*(176.04) = -3.43, *p* = 0.001, *d* = -0.45), and a shorter duration of OCD (*t*(169.12) = -4.35, *p* < 0.001, *d* = -0.58). Furthermore, these individuals showed a significantly shorter delay to recognition of OCD (Mann-Whitney-*U* = 7,283, *p* = 0.017, *r* = -0.14), a shorter delay to seeking professional help (Mann-Whitney-*U* = 5,410, *p* = 0.043, *r* = -0.13), and were more likely to currently receive CBT (Χ²(1) = 10.50, *p* = 0.001, Cramer-*V* = 0.20). Accordingly, individuals recruited via OCD Land exhibited significantly lower Y-BOCS (*t*(274) = -2.54, *p* = 0.012, *d* = -0.32) and PHQ-9 (*t*(179.13) = -2.26, *p* = 0.025, *d* = -0.30) scores as well as significantly increased AS (*t*(274) = 2.26, *p* = 0.025, *d* = 0.28) and quality of life (*t*(273) = 4.01, *p* < 0.001, *d* = 0.50). There were no significant group differences with respect to age, gender, rate of self-diagnosis, being currently medicated or ever having received CBT with ERP (all *p* > 0.05).

## Discussion

The present study comprehensively investigated help-seeking behavior, treatment barriers and facilitators, attitudes towards treatment options and access to gold-standard treatment in individuals with OCD from three different recruitment settings. By including people in- and outside the healthcare system, our findings fill the gap between representative epidemiological surveys and recent investigations in outpatient samples. To our knowledge, we conducted the largest such study in Germany. Overall, the rate of participants that had ever sought psychotherapeutic help for treating their OCD was high (88.4%), and with a mean of 5.15 years, the delay to seeking help was relatively low compared to previous studies [12]. In the present study, the mean delay to recognition of the symptoms as OCD was 5.58 years, indicating that many individuals with OCD did not receive a correct diagnosis at their first professional contact. Of those individuals without a professionally validated OCD diagnosis, 52.7% reported that they had sought professional help at some point, supporting the notion that OCD is not recognized/diagnosed by a substantial proportion of clinicians. As a longer delay to recognition of OCD was related to increased symptom severity, means that reduce this delay, such as accessible educational content, hold the potential to substantially improve the healthcare situation of those affected by OCD.

More severe OCD symptoms were also associated with a longer delay to seeking professional help, an increased number of contacted professionals before receiving psychotherapy, an increased number of treatments started until receiving ERP, more severe depressive symptoms, reduced acceptance, and lower quality of life, supporting the notion that system-related treatment barriers substantially contribute to a deteriorating course of OCD. While a longer illness duration alone was not related to an increased symptom severity, a longer duration of *untreated* or *inadequately treated* OCD accompanied by a prolonged, frustrating search for help may lead to a substantial worsening of the disease. Since a longer duration of untreated OCD has been linked to poor long-term treatment response in a recent meta-analysis [50], the promotion of access to early interventions is crucial.

Several previous studies have reported a substantial delay to diagnosis in OCD. For example, a recent study from the United States found a mean delay to diagnosis of 7.1 years in outpatients with OCD [51]. In a German outpatient sample, the mean delay to diagnosis was 12.78 years, plus an additional 1.45 years until the beginning of therapy [27]. To the best of our knowledge, no study has so far assessed the delay to recognition, i.e. the delay from OCD onset to the recognition of the disorder by the individual themselves. In our diverse sample that included individuals both in- and outside the healthcare system, we found a mean delay to recognition of 5.58 years. As this number reflects delay to self-diagnosis in cases where recognition preceded professional diagnosis, or where no professional diagnosis has yet been made, it appears plausible that the mean delay to recognition is shorter than previous reports of delay to diagnosis. Yet, it is still striking that individuals live more than five and a half years on average with distressing OCD symptoms before even finding a name for their problem.

Overall, participants rated educational websites such as OCD Land as the most important facilitators in recognizing their symptoms as OCD, followed by psychiatrists. The contribution of print media, YouTube videos and mental health channels on TikTok or Instagram was still rated higher than the contribution of general practitioners. Although most participants were recruited online and were hence biased to favor digital resources, 43.8% of participants from our outpatient clinic also stated that educational websites had a large or very large contribution to the recognition of their OCD. With regard to obtaining information about effective treatment options for OCD, the website OCD Land was again rated as the most helpful facilitator, followed by psychiatrists. Expectedly, individuals that were recruited via OCD Land were positively biased, but again, a substantial proportion of participants recruited via other access routes also endorsed the role of OCD Land as an important facilitator. Most participants did not have access to a specialized outpatient clinic for OCD, and those recruited via this access had mostly not started treatment, yet.

Regarding admission to gold-standard treatment, external treatment barriers were reported to have a stronger contribution on not receiving ERP than internal factors, with lack of knowledge about treatment options and contact points, therapists not performing ERP and too long waiting lists being the most common barriers. Consistent with previous studies [13], the most common internal barrier was the desire to handle the problem independently, followed by shame regarding the disease. Notably, fear of exposure was reported by very few individuals, and over 90% of participants endorsed (much or very much) that they would use CBT with ERP if there were no external barriers. The preference of CBT without ERP was substantially lower and similar to other treatment options such as psychodynamic therapy, indicating that participants were well-educated about evidence-based treatment for OCD across all recruitment groups. Still, a large proportion of individuals had not received ERP during CBT, so far. With roughly 49.5%, the prevalence of therapist-guided ERP was similar to previous studies [52, 53]. While we rate it as generally positive that 71.2% reported that ERP had at some point been assigned as homework, guidelines recommend that at least the first sessions of ERP should be performed in the presence of a therapist [54]. Of note, individuals had to start a median number of *Md* = 2 therapies until they received ERP treatment.

In accordance with previous studies from Germany [27] and the United States [51], younger individuals reported a significantly shorter delay to recognition of OCD and a shorter delay to seeking psychotherapy, which has been hypothesized to reflect an increased mental health literacy in younger generations due the use of online resources. We also observed a significant negative association between age and the assumption that psychotherapy would not work for the specific person, suggesting that younger individuals have more positive expectations towards psychotherapy. Interestingly, individuals who were recruited via OCD Land’s community forum and Instagram page reported a significantly shorter delay to recognition and delay to seeking help, although they were not significantly younger than participants recruited via other access routes. Furthermore, individuals recruited via OCD Land were more likely to currently receive CBT and appeared to be less strongly affected by the disease, as indicated by lower obsessive-compulsive and depressive symptom severity, increased acceptance and a higher quality of life. Given the cross-sectional nature of our study, it remains elusive whether the current receipt of CBT, the usage of OCD Land’s resources or distinct clinical characteristics such as an older age of onset and shorter illness duration contributed to this reduction in symptom severity. Future studies should systematically assess longitudinal effects of online self-help resources such as OCD Land on symptom severity and willingness to seek professional help.

With regard to symptom dimensions, taboo thoughts (60.9%) and checking (52.9%) were the most common, while symmetry/ordering symptoms were only reported by a few participants. Compared to previous studies [e.g. 55], the prevalence of taboo thoughts was substantially increased across all three recruitment groups, potentially indicating that aversive intrusive thoughts and associated mental compulsions are increasingly recognized as OCD and reported more openly. Washing/contamination symptoms were more common in individuals from the specialized outpatient clinic than in individuals that were recruited online, but not more common among individuals who had ever sought psychotherapy in general. Strikingly, individuals who had ever (currently or in the past) experienced washing/contamination symptoms were significantly more likely to have ever received ERP (therapist-guided or as homework) during CBT, suggesting that this symptom dimension is the clearest indicator for clinicians to conduct ERP. Although washing/contamination symptoms are substantially less common than other dimensions, they are among the most stereotypical OCD symptoms. More awareness should hence be raised that other symptom dimensions can be effectively treated with ERP, as well.

In line with previous research [56], individuals with current or past taboo (aggressive/sexual/moral/religious) thoughts were significantly more likely to report a professional diagnosis, to have ever sought psychotherapy for their OCD and to be currently treated with CBT. Since receiving the professional validation that the experienced thoughts, pictures or impulses are in fact an expression of OCD and do not indicate that the affected individual is a mass murderer or pedophile is extremely relieving for those affected, it seems plausible that individuals with taboo thoughts are more willing to approach a healthcare professional for diagnosis. Additionally, a professional diagnosis was associated with a younger age at first obsessive-compulsive symptoms, a younger age at OCD onset and a longer duration of the disease, suggesting that the longer an individual lives with OCD, the more likely they are to seek help. In younger individuals, help seeking and receiving a diagnosis may also be facilitated by parents.

Interestingly, individuals with self-diagnosed OCD did not significantly differ from individuals with a professional diagnosis regarding age, gender, OCD and depressive symptom severity, acceptance and quality of life, supporting the clinical validity of self-diagnosed OCD. Unlike other psychiatric disorders or syndromes that are at greater risk of over-diagnosis due to social media [34], OCD is still highly stigmatized and self-diagnosis is likely only applied when an individual experiences a substantial amount of suffering from their symptoms. Hence, data quality does not appear to be compromised in self-diagnosed individuals with OCD. In fact, assessing individuals with self-diagnosed OCD enables important insights into clinical and sociodemographic characteristics of those who have never approached a healthcare professional. Specifically, individuals who had never sought psychotherapy were more likely to be male and to have current order/symmetry symptoms. Compared to other symptom dimensions, order/symmetry symptoms have a stronger familiarity [57, 58], a higher comorbidity/overlap with tic disorders [59], a more pronounced association with feelings of incompleteness [60] and a higher rate of treatment resistance [61]. It may therefore be particularly important to tailor and validate treatment options for this specific subtype of OCD.

Unexpectedly, individuals without any comorbid anxiety disorder were more likely to be currently treated with CBT and to have ever received ERP to treat their OCD. At the same time, individuals with a self-diagnosed comorbid anxiety disorder (including social anxiety disorder) were less likely to be currently treated with CBT and to have ever received ERP, presumably because these individuals had a stronger wish to handle the problem on their own and were more avoidant. As the comorbidity between OCD and anxiety disorders is large, specifically addressing these individuals in educational articles and raising awareness among clinicians about combined ERP treatment for OCD and anxiety may contribute to an overall improvement of the healthcare situation of individuals with OCD.

With regard to medical treatment of OCD, the majority of participants had been medicated with SSRIs at some point. The single most important facilitator of medical treatment was a high level of suffering, followed by high expectations of effectiveness. Importantly, only a small proportion of participants endorsed psychotherapy not being (promptly) available as a motivator for taking medication. The most important reason for declining medication was the desire to handle the problem independently, followed by the fear of personality change. This fear was reported to have an even stronger impact than the fear of typical side effects (such as a dry mouth), suggesting that the dissemination of information on realistic and unrealistic side effects could be worthwhile.

### Limitations

Several limitations should be considered. First, all data are self-reported, including OCD diagnosis in recruitment groups 2 and 3 (self-diagnosis vs. professional diagnosis) and symptom severity. Yet, every participant reported a Y-BOCS score above the clinical cut-off value of 7 (despite the score being based on nine rather than ten items), and there was neither a significant difference in symptom severity between self-diagnosed and professionally-diagnosed individuals nor between the three recruitment groups. We are hence confident that using self-reported diagnoses is an efficient and ecologically valid approach to assessing a large sample of participants in- and outside the healthcare system. A second limitation concerns the calculation of the Y-BOCS total score. In order to cope with missing information from one item (due to a programming error), we employed an alternative computation method of OCD symptom severity proposed by Woody et al. [44], which addresses several limitations of the original Y-BOCS score (see Measures). The construct validity of the score we employed was also supported by positive correlations with depressive symptom severity, and negative correlations with quality of life and acceptance. The score thus represents a meaningful measure, though its comparability to other studies is slightly reduced. As a third limitation, we acknowledge that there are biases associated with non-probabilistic sampling and that the results may not generalize. For example, the large proportion of females and the relatively low rate of symmetry/ordering symptoms indicate that the sample is not fully representative. Moreover, individuals recruited online are inherently biased to favor online resources. Still, a large proportion of participants recruited via the outpatient clinic also reported positive attitudes towards educational websites and online self-help content, attenuating this limitation. Fourth, individuals who reported currently receiving CBT were at different stages of therapy, so that some individuals likely exhibited reduced symptom severity (towards the end of CBT treatment), while others showed higher levels of severity (at the beginning of CBT treatment). As a consequence, we did not observe any associations between currently receiving CBT and symptom severity.

## Conclusions

In sum, it appears that individuals with OCD are increasingly aware about ERP being the gold-standard treatment for their symptoms, and over 90% of participants reported to be willing to perform ERP if it was available. Delay to recognition of OCD and delay to seeking help still exceed 5 years on average, but were reduced in younger individuals, potentially reflecting increased mental health literacy and access to educational content online. Unfortunately, however, adequate treatment is not readily available, and many CBT therapists still do not offer ERP for OCD. Educational websites were identified as the most important facilitators in recognizing OCD and providing information on effective treatment options. Furthermore, lack of knowledge about treatment options and contact points was reported as the most common barrier to not seeking/receiving ERP-based treatment. Therefore, we propose the creation of an accessible online database of German CBT therapists specialized in the treatment of OCD, and to promote knowledge on ERP among therapists. Notably, access to ERP was facilitated in individuals with washing/contamination symptoms, which are among the most stereotypical expressions of OCD despite being less common than other symptom dimensions. Although our findings may not be fully representative, they stress the need for raising awareness about the diverse forms of OCD among both professionals and in the general population.

## Electronic supplementary material

Below is the link to the electronic supplementary material.

Supplementary Material 1

## Data Availability

The datasets analyzed during the current study are available from the corresponding author.

## Abbreviations

ADHD: Attention deficit hyperactivity disorder
AS: Acceptance Scale
CBT: Cognitive behavioral therapy
DGZ: Deutsche Gesellschaft Zwangserkrankungen e.V
ERP: Exposure and response prevention
OCD: Obsessive-compulsive disorder
PHQ-9: Patient Health Questionnaire-9
PTSD: Post-traumatic stress disorder
SSRIs: Selective serotonin reuptake inhibitors
Y-BOCS: Yale-Brown Obsessive-Compulsive Scale
