# Supplementary material for: Help-seeking behavior, treatment barriers and facilitators, attitudes and access to first-line treatment in German adults with obsessive-compulsive disorder
Source: BMC Psychiatry. 2025 Mar 11;25:235. doi: 10.1186/s12888-025-06655-0 (PMC11900428; doi:10.1186/s12888-025-06655-0)
Supplement: Supplementary file 1 — Supplementary Material 1 [file 12888_2025_6655_MOESM1_ESM.docx]

**Help-seeking behavior, treatment barriers and facilitators, attitudes and access to first-line treatment in German adults with obsessive-compulsive disorder – Supplement**

Katharina Bey, Severin Willems, Anna Lena Dueren, Alexandra Philipsen & Michael Wagner

**Questions to assess help-seeking behavior,** **treatment barriers and facilitators, attitudes towards different treatment options and access to ERP treatment**

1. What contribution did the following contact points and media make to you recognizing your symptoms as obsessive-compulsive disorder? (Scale: 1 (none) – 5 (very large); indicate for each item)
   General practioner; psychiatrist; educational websites (e.g. OCD Land); mental health channels on TikTok or Instagram; online platforms of major newspapers (e.g. ZEIT ONLINE); videos on YouTube; television programs; print media (books, newspaper articles, etc.)
2. What contribution have the following contact points and media made to obtaining information about effective treatment options for your OCD? (Scale: 1 (none) – 5 (very large); indicate for each item)
   General practitioner; psychiatrist; a specialist outpatient unit for OCD; the German OCD Foundation (DGZ); OCD Land; websites with therapy search function (e.g. therapie.de); friends/acquaintances/relatives
3. Have you ever *sought* psychotherapeutic treatment for your OCD? (yes/no)
4. If yes, how much time has passed between the onset of your OCD and the first time you sought treatment?
5. If yes, how many therapists have you contacted in the course of your search for treatment?
6. Have you ever *received* psychotherapeutic treatment for your obsessive-compulsive disorder? (yes, currently / yes, in the past / no; indicate for each item)
   Outpatient cognitive behavioral therapy; outpatient systemic therapy; outpatient psychodynamic therapy; outpatient psychoanalysis; (partial) inpatient cognitive behavioral therapy; (partial) inpatient psychodynamic therapy; other (partial) inpatient therapy
7. If you use/have used psychotherapy, was exposure and response prevention carried out as part of this? (yes/no; indicate for each item)
   With therapist; as homework
8. If yes, how many therapies did you start before you received exposure-based treatment?
9. What influence did the following factors have on the fact that you have not received/seen exposure-based treatment to date or in the past? (Scale: 1 (none) – 5 (very large); indicate for each item)
   Lack of knowledge about treatment options and contact points; waiting lists for therapists are too long; treatment services too far away; therapist did not offer exposure exercises; financial reasons; fear of exposure therapy; shame regarding the disease; desire to solve the problem alone; assumption that psychotherapy would not help me; I didn't want to take a treatment slot away from someone who might need it even more urgently
10. Have you ever received psychopharmacological treatment for your OCD? (yes, currently / yes, in the past / no / not sure; indicate for each item)
    SSRI (escitalopram, citalopram, sertraline, fluoxetine, fluvoxamine, paroxetine); SSRI plus atypical neuroleptic (risperidone, apiprazole, quetiapine, olanzapine, clozapine...); clomipramine
11. If yes, what influence did the following factors have on your decision to take medication for your OCD? (Scale: 1 (none) – 5 (very large); indicate for each item)
    High level of distress; high expectation of effectiveness; positive experiences with other medications; psychotherapy was not (promptly) available; other people motivated me to do so
12. If no, what influence did the following factors have on your decision not to take medication for your OCD? Scale: 1 (none) – 5 (very large); indicate for each item
    Fear of typical side effects (dry mouth, influence on libido...); fear of personality change; assumption that the medication will not help; shame or fear of stigmatization; desire to solve the problem alone; negative experiences with other medications; other people have advised against it
13. To what extent do you use the following self-help services to treat your OCD? (never / less than once a month / about once a month / about once a week / several times a week; indicate for each item)
    Local self-help group; online self-help group; self-help literature; online self-help material/content (on specific websites, social media or YouTube); online community/forums; self-help apps
14. If there were no barriers in the healthcare system, which therapy would you use to treat your OCD? (Scale: 1 (in no case) – 5 (in any case); indicate for each item)
    Exposure-based cognitive behavioral therapy; cognitive behavioral therapy without exposure; systemic therapy; psychodynamic therapy; psychoanalysis; medication with an SSRI; medication with an SSRI plus atypical neuroleptic; medication with clomipramine
15. If there were no barriers in the healthcare system, which treatment setting would you use (Scale: 1 (in no case) – 5 (in any case); indicate for each item)?
    Outpatient individual therapy; outpatient group therapy; in-person self-help group; self-help literature; online self-help content/material; online community/forum; self-help app
